# Integrating environmental effects in the benefit-risk assessment of therapeutic products: a proposal and example for sustainable health and healthcare

**DOI:** 10.3389/fdsfr.2024.1519142

**Published:** 2025-01-17

**Authors:** Emiliano Giovagnoni, Luisa Mattoli, Andrea Cossu, Vitalia Murgia

**Affiliations:** ^1^ Aboca S.p.A., Loc. Aboca, Sansepolcro, Italy; ^2^ Pediatric Unit, Department of Clinical, Surgical, Diagnostic and Pediatric Sciences, University of Pavia, Pavia, Italy

**Keywords:** benefit-risk assessment, environmental risk, one health, greener pharmacy, biodegradability, medical devices made of substances

## Abstract

To mitigate healthcare’s unintended effects, environmental risk data such as ecotoxicity and environmental contamination need to be considered by all stakeholders in the healthcare value chain. As decision-makers and educators, healthcare providers are in a unique position to make healthcare delivery more sustainable. However, current benefit-risk assessments of therapeutic products do not systematically include environmental risk data. The purpose of this paper was to review the literature and regulatory context and propose expanding benefit-risk assessments of therapeutic products to include an evidence-based evaluation of environmental impact to inform decision-making. Our findings indicate that environmental risk assessments need to be broadened to include aspects such as excipients, synergistic effects of contaminants, and risks to humans (e.g., drinking water). Concrete proposals to improve current regulatory guidelines for environmental risk assessment already exist. Open access databases on environmental risk of substances used in products for human consumption are available. The European Union Regulation on Medical Devices provides the basis for evidence-based approach to developing sustainable therapeutic products based on natural substances. Based on this, the here promoted decision scheme for healthcare providers (and other stakeholders) involves comparing the clinical safety and efficacy of therapeutic products; comparing environmental impact data; and then deciding. A case example involving the treatment of patients with gastroesophageal reflux and dyspepsia is presented. We provide suggestions for integrating persistence and ecotoxicity data into clinical practice. Expanding the benefit-risk assessment to include data on environmental impact during clinical decision-making is a way to achieve a healthier outcome for all.

## 1 Introduction

Current global trends in healthcare indicate that environmental issues no longer can or should be ignored. Innovation and the inclusion of environmental aspects in the development, evaluation, and delivery of healthcare treatments are urgently needed. Specifically, environmental aspects need to be included in benefit-to-risk assessments and this expanded criterion should be adopted by all stakeholders in the healthcare value chain. With regards to healthcare delivery, healthcare providers can have a particularly unique role in improving its sustainability as decision-makers who can (should) make an informed choice about treatment choices in consultation with their patients. Each decision can be a step toward increasing market demand for treatments that are more environmentally friendly and decreasing the entry of pollutants into water systems.

The paradox between the aim of healthcare to improve health and how the environmental impact of providing healthcare puts human health at risk must be addressed (Lenzen et al., 2020; Halonen et al., 2021; Mortimer and Pencheon, 2022). While advances in healthcare over the past decades have improved human health, healthcare has a considerable ecological footprint and contributes to anthropogenic changes such as loss of biodiversity and destabilization of planet’s ecosystems. Consequences of biodiversity loss and destabilized ecosystems that threaten human health include extreme weather events, air pollution, food and water insecurity, and infectious diseases (Romanelli et al., 2015).

Another specific example of how the unintended environmental effects of healthcare affect human health involves our microbiota and antibiotics (Prescott et al., 2022; World Health Organization, 2022). There is a growing evidence that excreted antibiotics contaminate water, soil, and vegetables and alter their microbial ecosystem, favoring the development and growth of resistant bacteria (Carvalho and Santos, 2016; Li et al., 2022; Grenni et al., 2018; Robles-Jimenez et al., 2021; Maghsodian et al., 2022; Symochko et al., 2023; Cycoń et al., 2019). Any antibiotic-induced perturbation of composition and diversity of the microbiota involves humans, animals, plants, and the environment as they continuously exchange microbiota, and often leads to an increase in phyla with a high content of antimicrobial resistance genes. In the case of humans, these resistant microbial strains and antibiotic residues enter the body via consumed crops and water; the resistant strains in particular could subsequently affect gut microbiota (Miller et al., 2016; Hirt, 2020; Flandroy et al., 2018; De Filippo et al., 2017). As the gut microbiome can be regarded physiologically as a human organ, when its structure is altered, the development of non-communicable diseases characteristic of “dysbiosis” is favored (De Filippo et al., 2017; Baquero and Nombela, 2012; Gill et al., 2006). Gut microbiota direct normal intestinal development and physiology; it also impacts the function of “diffuse systems” within the host such as those responsible for immunity, metabolism, and epigenetic modification of the genome, and “distant” organs such as the brain (Flandroy et al., 2018; Chow et al., 2010; de Vos et al., 2022). Thus, changes in the composition or abundance of the microbiota may lead to various diseases such as Alzheimer’s, colorectal cancer, nonalcoholic fatty liver disease, inflammatory bowel disease, hypertension, bipolar disorder, and obesity (de Vos et al., 2022; Clemente et al., 2012; Chen et al., 2021; Góralczyk-Bińkowska et al., 2022).

Clearly, safeguarding the environment is inseparable from safeguarding human health. The objective of our contribution is to propose expanding the benefit-risk assessment process of weighing benefits (positive effects) and risks (potential harm) of the various medical options for the diagnosis, prevention, or treatment of a medical condition with the additional consideration of a given medical option’s potential negative impact on the environment and public health. We first provide an overview of the evidence on trace pharmaceutics in the environment and the regulatory and scientific context to help frame our proposal for an expanded benefit-risk assessment. Then, we describe the new perspective offered by the European Union Regulation 2017/745 on Medical Devices (EU MDR) for therapeutics made from natural substances. We follow this with an example pertaining to the treatment of patients with gastroesophageal reflux disease (GERD) and dyspepsia to help illustrate the expanded benefit-risk concept. Last, we discuss strategies for implementing scientific evidence from ecotoxicity studies into clinical practice so that it can be used by healthcare professionals and patients in their decision-making process.

## 2 Context for expanding the benefit-risk assessment

### 2.1 Identifying relevant literature

A comprehensive search of articles was conducted in the following databases from inception to January 2024: PubMed, Scopus, Science Direct, and Google Scholar. Our search strategy was based on the concepts of benefit-risk assessment, environmental impact of healthcare and pharmaceuticals, medical devices made of substances (MDMS), One Health and planetary health, and the interrelationship between human health-gut microbiome-environment. Keywords used in various combinations included benefit-risk assessment, sustainability, environment, environmental sustainability, environmental impact, ecotoxicity, biodegradability, bioaccumulation, contamination, One Health, planetary health, gut microbiome/microbiota, (complex) natural substances, medical devices made of substances/substance-based medical devices, pharmaceuticals, excipients, additives, preservatives, parabens, and artificial sweeteners. We also reviewed the reference lists of included articles for other potentially relevant sources.

### 2.2 Evidence on trace pharmaceutics in the environment

The issue of the environmental risk must be taken in account in the light of the emerging concerns for human health due to water pollution by pharmaceutical and their metabolites (World Health Organization, 2012; Stockholm County Council, 2014; Norman Network, 2023). While it is beyond the scope of our contribution to provide a detailed analysis of the level of environmental drug pollution, we believe it is necessary to share some topical elements of this issue for a better understanding of the need to expand the benefit-risk assessment.

#### 2.2.1 Pharmaceutic residuals

Pharmaceutic residuals from the manufacturing process or related to patient use (i.e., patient urine and/or improper disposal of unused medication) enter the terrestrial and aquatic environments *via* wastewater (Moermond and de Rooy, 2022; Hofman-Caris et al., 2019; Molnarova et al., 2023). Unintended (additive) exposure in humans occurs through the drinking water supply and contamination of the food chain through root uptake of crops grown in soil irrigated with reclaimed wastewater or fertilized with biosolids from wastewater sludge (Miller et al., 2016; Yang et al., 2017; Dey et al., 2019; Paltiel et al., 2016). A review of studies conducted in Europe, Canada, the United States, and Brazil indicated that more than 80 pharmaceuticals, compounds, and drug metabolites have been detected up to the mg/L-level in sewage and surface waters (Heberer, 2002). Another review that analyzed over 200 studies of wastewater treatment plants (WWTPs) in Europe, Asia, and the United States found that the removal of pharmaceuticals varied between less than 15% to greater than 99% (Yang et al., 2017). Prescription categories of detected pharmaceuticals included analgesics, anti-inflammatory drugs, antibiotics, hormones, blood-lipid regulators, beta blockers, cytostatic drugs, and anti-epileptic drugs (Yang et al., 2017; Heberer, 2002). Variation in removal may be attributed to differences in removal efficiencies between treatment techniques implemented by WWTPs (Hofman-Caris et al., 2019; Dey et al., 2019).

The presence of pharmaceuticals and their residuals in the environment can have an adverse impact on nontarget organisms such as fish and amphibians, with a cascading effect on wider ecosystems (European Commission and Directorate General for Environment, 2020). Increasing amount of antibiotics into waters and soils creates a threat to all microorganisms in these environments, potentially accelerating the development, maintenance and spread of resistant bacteria and fungi (Cycoń et al., 2019; Kovalakova et al., 2020). Antibiotics can also be toxic to algae and cyanobacteria that form the base of the aquatic food chain, which in turn, can disrupt food webs (Kovalakova et al., 2020). Food webs can also be affected when a given pharmaceutical contaminant has different behavioral effects on prey and predators (Brodin et al., 2014).

#### 2.2.2 The case of excipients

As with pharmaceuticals, there is also growing concern about the environmental accumulation of excipients. Common examples are artificial sweeteners and parabens, which are a group of substances commonly used as preservatives. Although excipients have been deemed safe for human consumption, knowledge of their overall safety is still evolving (Naik et al., 2021; Belton et al., 2020; Schiffman et al., 2023). With regards to artificial sweeteners, for example, degradation of acesulfame-potassium by UV light exposure can result in trace products that may cause oxidative stress in fish (Belton et al., 2020). Crustaceans (*Daphnia magna*) and gammarids (*G. oceanicus, G. zaddachi)* have been found to demonstrate behavioral changes after exposure to sucralose (e.g., feeding and finding shelter, spatial orientation, identifying and avoiding predators, swimming speed and height), which in turn may impact the food web (Wiklund et al., 2014; Wiklund et al., 2012). In the common carp, exposure to environmentally relevant concentrations of sucralose led to significant increases in lipid peroxidation, hydroperoxide content, and protein carbonyl content in the muscle, gill, and brain. Significant changes in antioxidant enzymatic activity in muscle and gills were also observed (Saucedo-Vence et al., 2017).

With respect to parabens, their impact on humans and other organisms is still under debate. These endocrine-disrupting compounds are widely detected in the environment (e.g., water resources, soil and sediments, air and dust, and biota) and in the human body (e.g., serum, breast tumor tissue, breastmilk, and placental tissue). There is some evidence from human studies to suggest that even low concentrations of paraben can influence organism homeostasis (Błędzka et al., 2014). Recently, the toxicity of three parabens (methylparaben, propylparaben, and butylparaben) was tested in two fish (*Danio rerio* and *Cyprinus carpio*) and one amphibian models (*Xenopus laevis*). The results indicated that gene expression was affected for detoxification, sex hormone production, or cell stress signaling.

Metabolites of excipients can also be detrimental. A recently published article reported findings from a series of eight *in vitro* tests on sucralose-6-acetate, a metabolite and impurity from the manufacturing process. The results indicated that sucralose-6-acetate is genotoxic; *in vitro* exposure of intestinal epithelium (with absence of intestinal bacteria) to sucralose-6-acetate damaged the integrity of the intestinal barrier function; sucralose-6-acetate induced the expression of intestinal epithelial genes that are associated with oxidative stress, inflammation, and cancer; sucralose-6-acetate blocked two members of a key family of enzymes that play a key role in detoxification (Schiffman et al., 2023).

#### 2.2.3 Synergistic effects of contaminants

Another consideration is that synergistic effects of contaminants warrant proper attention. A recent study demonstrated that a synthetic hormone (17 alpha-ethinylestradiol, EE2) and a surfactant (sodium lauryl sulfate) not only had negative effects on mussel metabolism and oxidative status (*Mytilus galloprovincialis*) when acting alone but that the effect on behavior (i.e., valve closure) was greater in combination (Lopes et al., 2022). Furthermore, the findings from a proof-of-principle study involving a mixture of five steroid hormones on fish (i.e., fathead minnow) indicated that something can happen from “nothing”. That is, while each steroid hormone at low concentrations does not lead to a significant alteration in fish egg production on their own, simultaneous exposure of all five does have suppressive effects (Thrupp et al., 2018).

In sum, the long-term effect of additive exposure of trace quantities of pharmaceuticals and other chemicals to humans on development, disease resistance, and wellbeing is not known (Stockholm County Council, 2014). Nevertheless, it is clear risks exist and coordinated efforts to identify, measure, and mitigate these risks are needed. The entry of pharmaceutical pollutants into the water environment can be prevented with strategies to decrease input by producers, health professionals, and users and output loads by WWTPs (Moermond and de Rooy, 2022; Burns et al., 2018).

#### 2.2.4 Main databases available for consultation

To enhance the availability of information throughout the healthcare chain regarding the behavior of different types of products and substances in the environment, there are various databases in existence and under development. Some databases (e.g., ECOdrug (Verbruggen et al., 2018), PubChem (National Institutes of Health and PubChem, 2024), ECOTOXicology Knowledgebase (Olker et al., 2022), CompTox Chemicals Dashboard (United States Environmental Protection Agency, 2023), NORMAN Database System (Norman, 2024), ECHA Chemical Database (European Chemicals Agency, 2024)) are particularly useful for guiding research in this field and to understand the environmental fate of substances used for human consumption. Another, namely, the Swedish Pharmaceuticals and Environment database (Stockholm County Council, 2014; Wennmalm and Gunnarsson, 2009; Janusinfo, 2023) represents a collaborative effort of different healthcare stakeholders to promote transparency about the environmental risk of pharmaceuticals. It was developed with the aim to stimulate health professionals and patients to use medicines with a lower environmental impact, and in turn, stimulate the pharmaceutical industry to design and develop more environmentally friendly products. All these examples can be accessed by the public. An overview of the aforementioned databases is provided in Table 1.

**TABLE 1:** Overview of open access databases containing ecotoxicity and environmental risk data of substances used in products for human consumption.

Database	Description
ECOdrug	The ECOdrug is a recently launched research platform tool that contains information on the Evolutionary Conservation Of human Drug targets in over (currently) 600 eukaryotic species and provides information about legacy and innovative drugs and their protein targets across these species. Data on human drug targets for over 1000+ legacy drugs are available. Knowledge of drug target conservation can ensure that the most appropriate species are selected when performing environmental toxicological studies (Schiffman et al., 2023)
PubChem	PubChem is an open chemistry database at the NIH useful for general information about APIs and other chemicals. The database contains information on chemical structures, chemical and physical properties, biological activities, health, safety, toxicity data, and many others (Wiklund et al., 2014)
ECOTOXicology Knowledgebase (ECOTOX)	ECOTOX is a comprehensive knowledgebase managed by the United States EPA. It contains curated environmental toxicity data for over 12,000 chemicals and over 13,000 biological species. It includes over one million test records from over 50,000 references from the peer-reviewed and grey literature (Wiklund et al., 2012)
CompTox Chemicals Dashboard	This dashboard integrates data various sources such as PubChem, ECOTOX Knowledgebase, and the EPA’s computational toxicology research databases. It contains chemistry, toxicity, and exposure information for over one million chemicals, among which many APIs and excipients. Within the Dashboard, users can access high-quality chemical structures, experimental and predicted physicochemical properties, environmental fate and transport, and toxicity data, with links to relevant websites and applications. The ecological health information includes the lowest concentration of a chemical that produces an adverse effect for acute, chronic, and reproductive toxicity (Saucedo-Vence et al., 2017)
NORMAN Database System	This database system managed by the Norman Network is the reference platform for emerging contaminants in the environment. Using data input from contributors, it identifies the current most frequently discussed emerging substances and emerging pollutants. The database system comprises 11 web-based databases containing data on substances for suspect screening and prioritization, mass chromatograms, geo-referenced monitoring data, antibiotic resistant bacteria/genes in environmental matrices, data in indoor environment matrices, SARS-CoV-2 in sewage, mass spectra of emerging substances, data obtained with passive sampler, data obtained by analysis of environmental samples with bioassays, and ecotoxicity studies (Błędzka et al., 2014)
Pharmaceuticals and Environment Database	This database produced by Stockholm County Council classifies human medicines based on environmental hazard assessments (persistence, bioaccumulation potential, and ecotoxicity) and environmental risk (ratio between an API’s predicted environmental concentration and its predicted no effect concentration). To date, the system has classified more than 300 APIs, accounting for more than 50% of the volume of drugs in Sweden (Flandroy et al., 2018; Thrupp et al., 2018; Burns et al., 2018)
European Chemicals Agency (ECHA) Database	The EU’s largest public database on chemicals is maintained by ECHA. This database contains chemical information that is submitted by industry and generated from the EU’s regulatory processes, including that from the REACH Regulation. The database contains data on over 360,000 chemicals. A new platform for the database (ECHA CHEM) was launched January 2024 (Lopes et al., 2022)

Abbreviations: API, active pharmaceutical ingredients; ECHA, European Chemicals Agency; EPA, Environmental Protection Agency; EU, European Union; NIH, National Institutes of Health; REACH, Registration, Evaluation, Authorisation and Restriction of Chemicals.

### 2.3 Regulatory and scientific context

Transdisciplinary movements advocating a paradigm shift from public health to planetary health are gaining traction and there is increasing awareness of the One Health approach and the interconnectedness between the environment, animals, and humans (Halonen et al., 2021; Mortimer and Pencheon, 2022; World Health Organization, 2022; Monath et al., 2010; Marselle et al., 2021). In addition, various policies at the international (e.g., EU Green Deal (European Commission, 2023a), UN SDGs (United Nations Department of Economic, 2023)) and national (e.g., National Health Service Net Zero in the UK (National Health Service, 2020), Green Deal 3.0 in the Netherlands (Government of the Netherlands, 2022)) level support efforts to measure and mitigate the unintended environmental impact of the healthcare sector.

In recent years, various publications have proposed ways to improve the regulatory guideline for environmental risk assessments (ERAs) of human medicinal products issued by the European Medicines Agency (EMA) (Gunnarsson et al., 2019; Gildemeister et al., 2023). The existing guideline (currently under review) lists all the tests that must be performed as part of the ERA (European Medicines Agency and Committee for Medicinal Products for Human Use CHMP, 2006; European Medicines Agency and Committee for Medicinal Products for Human Use CHMP, 2018).

Furthermore, in April 2023, the European Commission released a proposal for revising the general pharmaceutical legislation and promote innovation, particularly for unmet medical needs, while reducing regulatory burden and the environmental impact of medicines (European Commission, 2023b). The European Commission has recognized that the pharmaceutical product lifecycle can have negative impacts on the environment and is taking some steps toward a stricter regulation in the process of authorization, distribution, and maintenance in the market of the drugs.

### 2.4 Greener pharmacy

There is growing awareness among healthcare professionals that they are in a natural and favored position to inform the public and advocate for change to make healthcare more sustainable. For instance, the Pharmaceutical Group of the European Union (PGEU) has identified community pharmacists as having a key role in improving public health by providing information on the availability of “greener” pharmaceuticals, which have comparable evidence for efficacy and safety. This is in addition to advising on proper handling, adherence, and disposal (Pharmaceutial Group of European Union, 2019). Their contribution along with that of regulatory authorities and the scientific community of both editors and investigators, and consumers are critical. To practice sustainable evidence-based healthcare, health professionals and consumers also need information on a treatment’s environmental impact to supplement information on safety and performance.

## 3 Discussion

### 3.1 Expanding benefit-risk assessment of therapeutic products to include environmental effects

A benefit-risk assessment is a comparative evaluation or weighing of benefits (positive effects) and risks (potential harm) of various medical options for treatment, prophylaxis, prevention, or diagnosis and is essential for decision-making. Benefit-risk assessments have evolved from unstructured, subjective approaches to structured frameworks that can be descriptive or quantitative. A detailed overview of different methodologies is provided, for example, by Kürzinger et al. (2020).

Regardless of the specificities and strengths and limitations of the different methodologies, all aim to increase the transparency of decision-making process across the life cycle of a given medical option (Kürzinger et al., 2020). Benefits are usually defined as the successful treatment of the condition for which the drug is indicated as well as patient-related outcomes such as functional improvement or improved quality of life or patient satisfaction (Kürzinger et al., 2020; Kumar, 2020). Risks consist of adverse drug reactions, which can range from minor symptoms (e.g., headache, nausea, or dry mouth) to rare, severe reactions (e.g., liver failure, anaphylactic reaction, or cancer) (Kumar, 2020). To assist in informed decision-making, a benefit-risk assessment should always be conducted relative to no therapy, standard treatment, or a relevant comparator (Council for International Organizations of Medical Sciences CIOMS, 1998).

Various healthcare stakeholders (i.e., regulators, pharmaceutical companies, healthcare providers and their professional organizations, patients and support networks) are potential end-users of a benefit-risk assessment. Multiple public and private initiatives to developing benefit-risk frameworks and tools exist, including the development of a common benefit-risk assessment for all stakeholders (Walker et al., 2009). EMA and FDA have recently adopted the multi-criteria decision analysis framework that uses both qualitative and quantitative data (Chisholm et al., 2021). However, regardless of the framework or tools used, data on environmental health risks are not included on a standard basis.

Our proposed expanded benefit-risk assessment of therapeutic products has two pillars: 1) weighing the evidence of clinical safety and efficacy; and 2) evaluation of biodegradability of all compounds (actives and excipients) and their ecotoxicity to organisms that comprise an ecosystem with damage at different levels, e.g., molecular, cell, or tissue damage; development damage; damage with behavioral effects (Naik et al., 2021; Wiklund et al., 2014; Wiklund et al., 2012; Błędzka et al., 2014; Gunnarsson et al., 2019). Thus, in the situation where two or more treatments are comparable in terms of their clinical benefits and risks, then comparing the environmental data can help steer the choice to the more sustainable option. In the situation where the treatment option with a more favorable clinical benefit-risk profile has a greater environmental impact, mitigating strategies can then be considered and implemented.

It should be noted that although we have framed our proposal with the aim to affect the healthcare professionals and patients during their decision-making process, we also call on regulatory authorities and the scientific community to support this effort and contribute. When there is an environmental risk with a therapeutic product that has health benefits, then mitigating strategies need to be identified, developed, and implemented to minimize the impact on the planet. A broader benefit-risk assessment that includes environmental effects will help ensure we do not continue to turn a blind eye to the unintended effects of healthcare (Smith et al., 2022).

### 3.2 New perspectives: the use of natural substances in therapy

Therapeutic products based on natural substances, which have been developed within an allopathic and evidence-based practice approach, offer new perspectives for sustainable healthcare in line with the proposed expanded risk-benefit concept. The basis for this opportunity is provided, thanks in part to the certification of the EU MDR (European Parliament and Council of the European Union, 2017). In addition to reforming the medical device approval and post-marketing evaluation where clinical data are essential for demonstrating or confirming medical device conformity with relevant general safety and performance requirements, this regulation provides new provisions (i.e., specific classification rules and requirements) for MDMS. Of utmost importance, the EU MDR allows the certification of natural products whose mechanism of action is not linked to the specific interaction of an active molecule and its biological target (commonly a receptor), but rather on the interaction of the entire complex matrix.

One example involves the treatment of functional constipation in young children (age range: 6–48 months). Findings from a randomized controlled trial (RCT) comparing the CE-marked Promelaxin microenema made of 100% natural substances (Melilax, a class IIb MDMS) with the standard first-line treatment polyethylene glycol (PEG) 4,000 indicated that Promelaxin was non-inferior and that safety was comparable. Though only considered hypothesis-generating, the analysis of microbiota data suggested that Promelaxin may have a potentially lower impact on microbiota than PEG (Strisciuglio et al., 2021).

Below we provide a more extensive example of how a therapeutic product made of 100% natural substances was evaluated in terms of clinical safety, efficacy, and environmental risk using an evidence-based approach. We then use it to illustrate how the concept of an expanded risk-benefit approach can be applied to identify products that are safer for humans and the environment without depriving patients of necessary therapy.

#### 3.2.1 Case example: poliprotect versus omeprazole

This example involves a mucosal protective agent (MPA) made of 100% natural substances (NeoBianacid, Aboca, Sansepolcro, Italy) and certified as class III medical device according to the EU MDR. This MPA is composed of Poliprotect (polysaccharide fraction from *Aloe vera*, *Malva sylvestris*, and *Althea officinalis*; minerals limestone and nahcolite) and a flavonoid fraction (from *Glycyrrhiza glabra* and *Matricaria recutita*). First, this MPA was compared to the standard of care (i.e., a proton-pump inhibitor, PPI; namely, omeprazole) in a double-blind, double-dummy, multicenter RCT (Corazziari et al., 2023). Then, the biodegradability of the two substances was investigated with an experiment conducted according to Organisation for Economic Co-operation and Development (OECD) test guidelines (OECD, 1992).

The efficacy and safety of the MPA were evaluated as compared with a standard dose of PPI (omeprazole 20 mg) in 273 endoscopy-negative patients with heartburn, a typical symptom of GERD and/or epigastric pain or burning (i.e., epigastric pain syndrome, EPS). In short, the primary efficacy endpoint was between-group comparison for the severity of heartburn and/or epigastric pain or burning from baseline to day 14 on a 100 mm visual analog scale. Secondary efficacy endpoints included comparison for change in symptom severity at earlier and later time points; use of rescue medicine; change from baseline in Quality of Life Index and Gastrointestinal Symptom Rating Scale score. Gut microbiota change was also assessed. Clinical safety was assessed by comparing number, proportion, and severity of total adverse events (AEs) as well as treatment related AEs, either overall or by System Organ Class, observed in each treatment group, and by means of routine blood and urine testing before and after treatment (Corazziari et al., 2023).

#### 3.2.2 Proposed decision scheme

**FIGURE 1:**
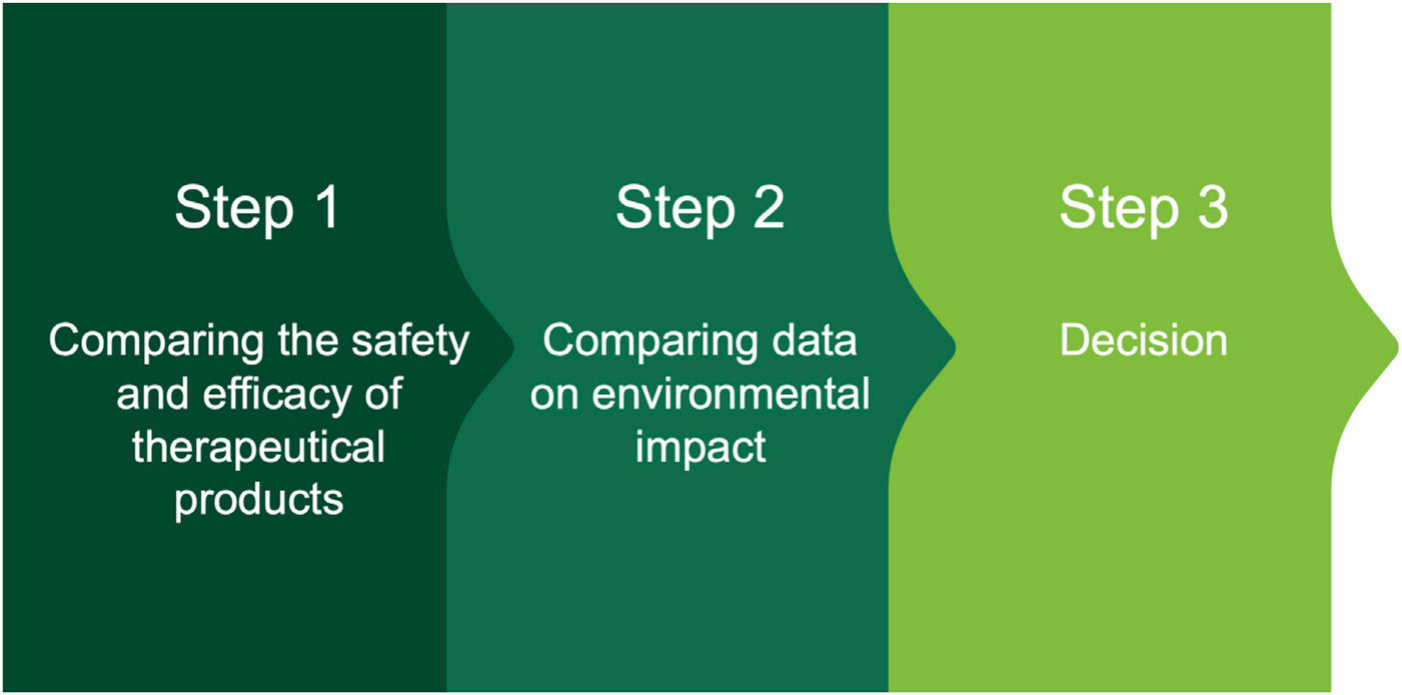
Decision scheme to support the choice of therapeutic solutions based on an assessment of safety, efficacy, and environmental impact.

Based on preceding discussion, it is possible to hypothesize the following decision scheme to support the choice of therapeutic solutions in a prudent manner. The steps of the scheme are shown in Figure 1 and are summarized below.

Step 1: Comparing the efficacy and clinical safety of Poliprotect with omeprazole

In terms of clinical benefits, the MPA proved non-inferior to a standard dose of omeprazole for symptom relief, indicating MPA as a valid alternative treatment to PPI in managing EPS and heartburn in the absence of esophageal mucosal lesions. Also, the initial benefit obtained with daily MPA could be maintained with on-demand therapy. With regards to safety, no relevant AEs were reported in either group. Lastly, as for gut microbiota analysis, the use of PPI, as compared to MPA, was associated with a significantly higher over time dissimilarity due to oral cavity genera enrichment (Corazziari et al., 2023).

Step 2: Comparing data on environmental impact of Poliprotect versus omeprazole

A study using a suspect screening analysis to improve untargeted and targeted UHPLC-qToF approaches evaluated the biodegradability of Poliprotect and omeprazole. Results at day 28th indicated that Poliprotect is readily biodegradable and that omeprazole is not, aligning with previous independent environmental research data (IVL Swedish Environmental Research Institute, 1991). In the case of omeprazole, 11 transformation products were identified. Findings from untargeted analysis and suspects screening indicating incomplete mineralization of omeprazole were confirmed by targeted analysis (Mattoli et al., 2024). The toxicity on humans of omeprazole degradation products, to our knowledge, has resulted only from predictive *in silico* analyses, which were related to omeprazole forced degradation products (Shankar et al., 2019) and which may not correspond to those produced under normal conditions. Human urinary omeprazole metabolites may accumulate in surface water and wastewater, placing aquatic organisms at risk (Boix et al., 2014; Chiriac et al., 2024).

Step 3: Decision

Combining the evidence from steps one and two supports the choice for prescribing Poliprotect as opposed to omeprazole in patients with heartburn and functional painful dyspepsia. That is, Poliprotect is equally effective compared with omeprazole in terms of symptomatic relief and thanks to its biodegradability, its use will not contaminate the environment. Conversely, PPI remains the first-choice therapy of patients with erosive esophagitis and dyspeptic symptoms due to gastroduodenal lesions, provided the absence of evidence supporting a comparable safety and clinical efficacy for alternative therapy options.

### 3.3 Integrating persistence and ecotoxicity data into clinical practice

Addressing the environmental impact of healthcare is a complex issue, with multiple stakeholders, uncertainties, and no single, easy solution. As such, successful mitigation of healthcare’s unintended environmental effects requires all stakeholders in the value chain to be involved and contribute where they can contribute (Moermond and de Rooy, 2022), and that robust scientific data are available for evidence-based and data-driven decision-making. With our contribution, we would like to affect the healthcare professionals during their decision-making process with regards to which therapeutic options they will wish to prescribe or recommend, and with what sustainability implications. Namely, that when evidence for clinical performance and safety are comparable, they should also consider environmental safety. Ideally, they also involve their patients in this process. By doing so, together they can contribute to the solution by decreasing the input of pharmaceuticals into the wastewater system.

Additional strategies to facilitate the integration environmental issues of healthcare into daily clinical practices include the following:

- educating patients on limiting self-medicating purchases, reducing the storage of excessive stocks of medicines at home, and proper disposal practices of unused and unwanted pharmaceuticals;
- including environmental aspects of pharmaceuticals in professional practice guidelines and information materials for healthcare professionals and patients. This may be facilitated by including environmental/ecotoxicity specialists in the team that is developing and updating such documents;
- embedding One Health, planetary health, and environmental aspects of medical products in the training of all healthcare professionals and continuing education programs (Pharmaceutial Group of European Union, 2019);
- leveraging informatics, for example, by integrating environmental data into online tools for clinicians such as electronic medication management systems (Smith et al., 2022). For example, in cases where there are two or more therapeutic options with comparable efficacy and safety, the default recommendation or prescription may be set to the one with a smaller environmental impact. When comparative data of different therapeutic options are not available, then the program could be set to alert healthcare providers to discuss mitigating strategies such as urine collection (such in the case of oncologic drugs that must be inactivated before disposal in the sewer system) or proper disposal with their patients.

## 4 Conclusion

While ensuring that every patient gets the treatment they deserve, it is essential to safeguard the health of the entire human population by protecting the environment and biodiversity. The case example of MPA illustrates that products with comparable performance and human safety can have different environmental impacts. As prescribers and patient educators, healthcare professionals can have a critical role in increasing the market demand for environmentally friendly products and preventing the entry of pharmaceutical residuals into water systems. In conclusion, we propose that expanding the benefit-risk assessment to include data on environmental impact during clinical decision-making is a way to achieve a healthier outcome for all.

## Abbreviations

API, active pharmaceutical ingredient; EMA, European Medicines Agency; EPA, Environmental Protection Agency; EPS, epigastric pain syndrome; EU MDR, European Union Regulation 2017/745 on Medical Devices; GERD, gastroesophageal reflux disease; MDMS, medical device made of substances; MPA, mucosal protective agent; OECD, Organisation for Economic Co-operation and Development; PEG, polyethylene glycol; PGEU, Pharmaceutial Group of European Union; PPI, proton-pump inhibitor; RCT, randomized controlled trial.
